# Preventable Blindness

**Published:** 1909-08-07

**Authors:** 


### "PREVENTABLE BLINDNESS."

### REPORT OF THE COMMITTEE

Appointed at the International Conference on the Blind, Held at Manchester in July, 1908.

The Hon. Secretary of the Committee on Preventable Blindness, Mr. C. M. Collingwood, of Exeter (Superintendent of the West of England Institution for the Blind), has sent the following report to us for publication :—

"The subject of the prevention of the disease which so frequently causes blindness in newly-born infants is one that for many years has engaged the attention of all whose work has brought them into contact with the blind. At every conference that has been held during recent years relative to the welfare of the blind, whether at home or abroad, attention has been called to the urgency for taking satisfactory measures for the checking of this disastrous cause of blindness.

"The medical libraries contain a host of papers and books which have treated of this disease, and of the blindness consequent thereon. Recent English literature has been summarised in the report of the British Medical Association committee (*British Medical Journal*, May 8, 1909), to which reference will be made further. The gist of these papers is to the effect :—(1) That more than one-third of the blindness found in British blind schools is due to this one cause—infants born with good eyes are blinded by the disease within a few days of their birth. (2) The risk of blindness is entirely preventable by prompt and efficient treatment.

"To this end it is now almost universally agreed, by both medical men and laymen cognisant with the subject, that the disease known as 'the ophthalmia of the new-born' should be scheduled as compulsorily notifiable. And it is believed that by such action this appalling source of misery and helplessness would be checked. In spite, however, of these urgent recommendations, no action has been taken by the British Public Health Authorities, nor have these authorities, so far, availed themselves of their permissive powers to make the disease compulsorily notifiable.

"At the International Conference on the Blind, held in Manchester in July, 1908, it was held that no sufficient steps had been taken to give publicity to the urgency of the matter. A representative committee was therefore appointed to consider the subject fully, and to make such recommendations as they might deem not only advisable, but as likely to be of practical utility. At the first meeting of this Committee two ophthalmic surgeons were co-opted as members of, and advisers to, the Committee :—Mr. Robert N. Hartley (Hon. Surgeon, Leeds United Institution for the Blind and Deaf and Dumb), and Mr. N. Bishop Harman (oculist to the London County Council Blind Schools). These gentlemen were expected to advise on matters relating to the North and South of England respectively. During the sittings of this Committee, another committee, instituted by the British Medical Association, has had this same matter under review. The two committees have acted upon quite independent lines, and the fact that they have arrived at similar conclusions, and make virtually the same recommendations, adds greatly to the weight of their recommendations.

"The following are the recommendations of the Committee appointed by the International Conference :—1. That in the opinion of this Committee the adoption by the Public Health Authorities of the Early Notification of Births Act is urgent. 2. That in the opinion of this Committee the disease known as the 'Ophthalmia of the New-Born' should be added to the list of diseases compulsorily notifiable under the powers of the Infectious Diseases (Notification) Act of 1889. 3. That in the opinion of this Committee :—(*a*) More definite teaching should be given to midwives on the seriousness of eye disease in children; and (*b*) they suggest that the Central Midwives Board should issue more stringent instructions on the danger of 'whites' in lying-in women.

"With a view to obtaining more exact knowledge of the incidence of 'blindness' in subsequent census returns, the Committee recommend that the Registrar-General should be requested to define and classify 'blindness' in his schedules in some such way as the following :—1. Stone Blind, *i.e.* the individual has no power to see the movement of fingers before the eyes. 2. Partially Blind, *i.e.* in the case of (i.) *Children.* Those who have not sufficient sight (even with the aid of glasses) to be taught in an ordinary school. (ii.) *Adults.* Those who have not sufficient sight (even with the aid of glasses) to earn a living by ordinary means.

"In conclusion, the Committee desire to emphasise the fact that the question is at the present juncture, and is likely to be in the future, of much greater importance than in the past. For the introduction and extension of the operation of the Employers' Liability Act is seriously affecting, and is likely to still more seriously affect, the earning powers of adult workers with defective vision."

The Report is dated June, 1909, and signed by the Chairman, Mr. Henry J. Wilson, and the other eight members of the Committee.

THE HOSPITAL August 7, 1909.

